# Escalating Use of Antimicrobial Agents in Tertiary Hospitals: A Balancing Act in Preventing Hospital-Acquired infections

**DOI:** 10.31729/jnma.v63i292.9252

**Published:** 2025-12-31

**Authors:** Lakshya Nehal Samineni

**Affiliations:** 1 College of Medical Sciences, Bharatpur, Nepal

**Dear Editor,**

The escalating reliance on antimicrobial medications within tertiary care institutions, especially towards the prevention of hospital-acquired infections (HAIs), demand urgent attention and rational debate. These medicines have undoubtedly played an invaluable role in reducing infection-related morbidity and mortality among critically ill patients. However, their growing prophylactic and empirical use has led to serious consequences, Particularly the acceleration of antimicrobial resistance (AMR).

Hospital settings, and intensive care units ICUs in particular, are recognized as breeding grounds for HAIs due to frequent use of invasive devices, compromised host immunity, and the presence of multidrug-resistant pathogens. In such environments, prophylactic antibiotic use often becomes a routine precautionary practice. Nevertheless, data suggests that a significant proportion of such use in inappropriate or unwarranted, and 30-60% of antibiotic prescriptions in ICUs have been estimated to be not adequately justified.^1^

These global concerns are reflected in the Nepalese context as well. Recent evidence from Nepal highlights an increasing trend in use of antibiotics, particularly those chategorized under the Watch group antibiotics by the World Health Organization (WHO) such as piperacillin/tazobactam and vancomycin. This trend, driven largely by empirical therapy rather than targeted therapy.^2^ Moreover, the pandemic of COVID-19 excaberated the issue as clinicians were faced with uncertain or questionable diagnoses, often resorted to broad-spectrum antibiotics in non-bacterial conditions.^3^

While prophylactic use of antimicrobials is occasionally indicated- e.g., for surgical or immunocompromised patients- the indiscriminate and prolonged use without strict infection control practices can paradoxically increase the risk of HAI by the growth of resistance microbes.^4^ Furthermore, poor stewardship infrastructure, limited access to prompt teeing, and limited use of culture data also promote inappropriate use in limited-resource settings.^5^

It is against this backdrop that it is important that tertiary care hospitals place great emphasis on antimicrobial stewardship programs (ASPs), reinforce infection prevention and control (IPC) practices, and implement electronic surveillance systems to track antibiotic use and resistance patterns. In addition, compliance with WHO’s AWaRe (Access, Watch and Reserve) classification and Essential Medicines Lift must be institutionalized to guide rational prescribing.

As antimicrobial resistance continues to threaten global health security, proactive transformation in prescribing culture, clinician education, and policy implementation must be the cornerstone of HAI prevention without compromising the future efficacy of antibiotics.
